# Pan-Cancer Characterization of Intratumoral Autonomic Innervation in 32 Cancer Types in the Cancer Genome Atlas

**DOI:** 10.3390/cancers14102541

**Published:** 2022-05-21

**Authors:** Jeff F. Zhang, Haiyang Sheng, Jianhong Chen, Hemn Mohammadpour, Sung Jun Ma, Mark K. Farrugia, Shipra Gandhi, Elizabeth G. Bouchard, Anurag K. Singh, Elizabeth A. Repasky, Thaer Khoury, Christine B. Ambrosone, Song Yao

**Affiliations:** 1 Jacobs School of Medicine and Biomedical Sciences, University at Buffalo, Buffalo, NY 14263, USA; jeffzhan@buffalo.edu; 2 Department of Cancer Prevention and Control, Roswell Park Comprehensive Cancer Center, Buffalo, NY 14263, USA; haiyang.sheng@roswellpark.org (H.S.); jianhong.chen@roswellpark.org (J.C.); elizabeth.bouchard@roswellpark.org (E.G.B.); christine.ambrosone@roswellpark.org (C.B.A.); 3 Department of Biostatistics, University at Buffalo, Buffalo, NY 14263, USA; 4 Department of Immunology, Roswell Park Comprehensive Cancer Center, Buffalo, NY 14263, USA; hemn.mohammadpour@roswellpark.org (H.M.); elizabeth.repasky@roswellpark.org (E.A.R.); 5 Department of Radiation Medicine, Roswell Park Comprehensive Cancer Center, Buffalo, NY 14263, USA; sungjun.ma@roswellpark.org (S.J.M.); mark.farrugia@roswellpark.org (M.K.F.); anurag.singh@roswellpark.org (A.K.S.); 6 Department of Medicine, Roswell Park Comprehensive Cancer Center, Buffalo, NY 14263, USA; shipra.gandhi@roswellpark.org; 7 Department of Pathology, Roswell Park Comprehensive Cancer Center, Buffalo, NY 14263, USA; thaer.khoury@roswellpark.org

**Keywords:** cancer, innervation, TCGA, autonomic nervous system

## Abstract

**Simple Summary:**

There have been growing interests in the roles of intratumoral innervation of the autonomic nervous system (ANS) as a mechanism linking psychosocial stress, β-adrenergic signaling pathways, and poor cancer outcomes, and a potential target for therapeutic purpose. Our current knowledge is being limited by the few cancer types where intratumoral ANS have been studied; it remains to be determined the extent of this mechanism existing in different cancer types. Our study provided the first pan-cancer characterization of intratumoral innervation across 32 cancer types, and further, their relationships with tumor histopathological and molecular characteristics and survival outcomes. We found wide variations in intratumoral ANS expression both within and across cancer types. The association of ANS signatures with tumor histopathological characteristics and survival outcomes also varied by cancer type. Our findings suggest that the potential benefits of cancer therapies targeting β-adrenergic receptor-mediated stress signaling pathways are likely dependent on cancer type.

**Abstract:**

Over the past two decades, multiple studies have demonstrated the important role that the autonomic nervous system (ANS) plays in tumorigenesis and cancer progression. However, the mechanisms by which this process occurs have only recently begun to be elucidated. Further, the extent of autonomic innervation in various cancer types and its effects on tumor molecular, immunological, and histopathological features, as well as on patient outcomes, are not yet fully characterized. In this study, we analyzed intratumoral ANS gene expression signatures, including overall intratumoral neuron growth and sympathetic and parasympathetic markers, across 32 cancer types using tumor transcriptomic and clinical annotation data available from The Cancer Genome Atlas (TCGA). Our analysis revealed wide variations in intratumoral ANS expression both within and across cancer types. The association of ANS signatures with tumor histopathological characteristics and survival outcomes also varied by cancer type. We found intratumoral ANS expression to be commonly correlated with angiogenesis, TGF-β signaling, and immunosuppression in the tumor microenvironment of many cancer types, which provide mechanistic insights into the involvement of intratumoral innervation in cancer development and progression. Our findings suggest that the potential benefits of cancer therapies targeting β-adrenergic receptor-mediated stress signaling pathways are likely dependent on cancer type.

## 1. Introduction

Prior studies have suggested that chronic psychosocial stress may be associated with tumor metastasis and worse long-term cancer prognosis [1], but the underlying biological mechanisms through which these adverse outcomes may occur are poorly understood. One key function of the central nervous system (CNS) is the response to perceived threats in the outside world and activation of the sympathetic nervous system (SNS) through adrenergic receptors that trigger an acute “fight or flight” stress response. It has been proposed that in states of chronic stress, persistent sympathetic activation and increased release of norepinephrine into the tumor microenvironment (TME) may trigger metabolic changes in vascular endothelial cells which promote angiogenesis and increase tumor vascularization [2]. Elevated levels of catecholamines also activate β2-adrenergic receptor (ADRβ2)-mediated cyclic adenosine monophosphate (cAMP) signaling pathways in tumor cells [3], which subsequently produce changes in the extracellular architecture that allow for further tissue invasion and metastasis by cancer cells. In addition, β2-adrenergic receptor activation inhibits the egress of CD8^+^ T-cells from tumor-draining lymph nodes [4,5], thus impairing the development of an effective antitumor immune response.

Recent studies have shown that tumor cells are themselves capable of producing neurotrophic factors, including nerve growth factor (NGF), brain-derived neurotrophic factor (BDNF), neurotrophin-3 (NT3), and neurotrophin-4/5 (NT4/5), to facilitate the infiltration of nerve fibers into their local TME and support their growth and invasion [6]. Consequently, β-adrenergic sympathetic innervation of tumors has been associated with increased tumor aggressiveness [6] and cancer progression in some cancer types [7], while animal models demonstrate that axotomy of sympathetic inputs to tumors suppresses their growth [8]. Parasympathetic stimulation in prostate tumors has also been shown to enhance tumor invasion of nearby lymph nodes in a reversible process that is inhibited by treatment with scopolamine, a nonselective muscarinic antagonist [8]. However, in breast tumors, stimulation of sympathetic nerves correlated with tumor progression and increased expression of immune checkpoint molecules, promoting an immunosuppressive TME, but subsequent parasympathetic activation conversely slowed the growth of cancer cells and decreased expression of immune checkpoint molecules [9]. These prior studies suggested that the roles of intratumoral innervation may vary by cancer types, yet the extent of autonomic innervation and its effects on tumor biology and patient outcomes across common cancer types have not been fully characterized.

We herein analyzed three ANS gene expression signatures in tumor samples from >10,000 patients across 32 cancer types in The Cancer Genome Atlas (TCGA) to determine the relative level of overall intratumoral neuron growth (TNG), SNS, and PNS innervation. We then analyzed the correlation of these signatures with selected tumor molecular and immunological features, as well as cancer histopathological features and survival outcomes.

## 2. Methods

### 2.1. Data Source

Normalized tumor RNA-sequencing data and clinical annotation data from 10,052 patients diagnosed with 32 cancer types available in The Cancer Genome Atlas (TCGA) (version 1.16, release date: 17 April 2019) were downloaded from the Genomics Data Commons (GDC) data portal.

### 2.2. Definition of ANS Gene Expression Signatures

Marker genes for the three ANS expression signatures included the following: *Nerve Growth Factor (NGF)* and *Neurofilament Light Chain (NEFL)* for overall intratumoral neuron growth (TNG); *Tyrosine Hydroxylase (TH)*, *Adrenoceptor Beta 2 (ADRB2)*, *Adrenoceptor Beta 3 (ADRB3)*, and *Brain-Derived Neurotrophic Factor (BDNF)* for sympathetic nervous system (SNS); and *Vesicular Acetylcholine Transporter (VACHT or SLC18A3)*, *Neuronal Nitric Oxide Synthase (NNOS or NOS1)*, and *Vasoactive Intestinal Peptide (VIP)* for parasympathetic nervous system (PNS). For each signature, the average levels of the selected marker genes were computed using normalized and log2-transformed RNA-seq data.

### 2.3. Selected Tumor Molecular and Immunological Features

To determine their relationships with autonomic innervation, data of selected tumor molecular and immunological features were derived either from the literature or from the RNA-seq data, which included angiogenesis [10], hypoxia [11], transforming growth factor- β (TGF-β) [12], cytolytic activity [13], leukocyte fraction [12], lymphocyte fraction [12], regulatory T-cell (T-reg) fraction [12], and expression levels of Programmed Cell Death 1 (PDCD1 or PD-1) and Programmed Cell Death 1 Ligand 1 (CD274 or PD-L1).

### 2.4. Statistical Analysis

Pearson correlation tests were used to test the relationships between ANS signatures and selected molecular and immunological features within each cancer type, where data were available from at least 100 cases. ANOVA was used to test differences in ANS signatures by clinicopathological variables, with *p*-values for trends derived from linear regression. For analysis of all-cause mortality, Kaplan–Meier curves were constructed for high vs. low levels of ANS signatures as determined by the median within each cancer type. Hazard ratios (HRs) and 95% confidence intervals (CIs) were estimated using Cox proportional hazard regression. All analyses were performed in R 4.1.1. Multiple comparison error was corrected by Bonferroni method to adjust the *p*-value cutoff by the number of tests performed.

## 3. Results

### 3.1. Pan-Cancer Characterization of ANS Signatures

**Figure 1:**
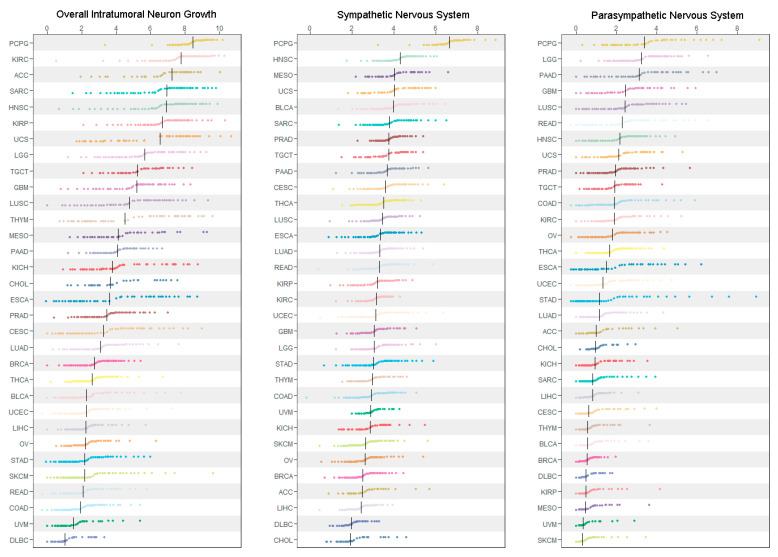
Pan-cancer characterization of autonomic nervous system gene expression signatures.

Gene expression signatures of overall TNG, SNS, and PNS across the 32 cancer types we surveyed were derived from TCGA tumor transcriptomic data. These results are summarized in Figure 1. Large intra- and inter-cancer type variations were observed for each signature. Pheochromocytoma and paraganglioma (PCPG), two neuroectodermal tumors of the adrenal medulla known for abnormally elevated sympathetic activity, had the highest levels of expression for all three ANS signatures. Other cancer types that were consistently ranked within the top 10 highest levels of expression for all three signatures included squamous cell carcinoma of the head and neck (HNSC), uterine carcinosarcoma (USC), and testicular germ cell tumor (TGCT). Brain tumors, including low grade glioma (LGG) and glioblastoma multiforme (GBM), expressed strong TNG and PNS signatures, but relatively weak SNS signatures. Sarcoma (SARC) had high expression of TNG and SNS gene markers but not PNS signature. Breast cancer (BRCA), the most common cancer in women, was ranked low on all of the three signatures, whereas prostate cancer (PRAD), the most common cancer in men, had a strong expression of both SNS and PNS signatures. At the lower end of the spectrum of ANS signatures were melanoma (uveal melanoma (UVM), skin cutaneous melanoma (SKCM)) and diffuse large B-cell lymphoma (DLBC).

Relative levels of gene expression signatures (*x*-axis) are calculated as the average levels of the selected marker genes using normalized and log2-transformed RNA-seq data, which are then used for ranking and plotting and coded in different colors according to the TCGA coloring schema. Vertical black lines indicate median levels within each cancer type, which are used to rank cancer types into high to low levels of gene expression. TCGA cancer symbols are as follows: ACC, adrenocortical carcinoma; BLCA, bladder urothelial carcinoma; BRCA, breast invasive carcinoma; CESC, cervical squamous cell carcinoma and endocervical adenocarcinoma; CHOL, cholangiocarcinoma; COAD, colon adenocarcinoma; DLBC, diffuse large B-cell lymphoma; ESCA, esophageal carcinoma; GBM, glioblastoma multiforme; HNSC, head and neck squamous cell carcinoma; KICH, kidney chromophobe; KIRC, kidney renal clear cell carcinoma; KIRP, kidney renal papillary cell carcinoma; LGG, brain low grade glioma; LIHC, liver hepatocellular carcinoma; LUAD, lung adenocarcinoma; LUSC, lung squamous cell carcinoma; MESO, mesothelioma; OV, ovarian serous cystadenocarcinoma; PAAD, pancreatic adenocarcinoma; PCPG, pheochromocytoma and paraganglioma; PRAD, prostate adenocarcinoma; READ, rectum adenocarcinoma; SARC, sarcoma; SKCM, skin cutaneous melanoma; STAD, stomach adenocarcinoma; TGCT, testicular germ cell tumors; THCA, thyroid carcinoma; THYM, thymoma; UCEC, uterine corpus endometrial carcinoma; UCS, uterine carcinosarcoma; UVM, uveal melanoma.

### 3.2. Correlations between ANS Signatures and Tumor Molecular and Immunological Features

**Figure 2:**
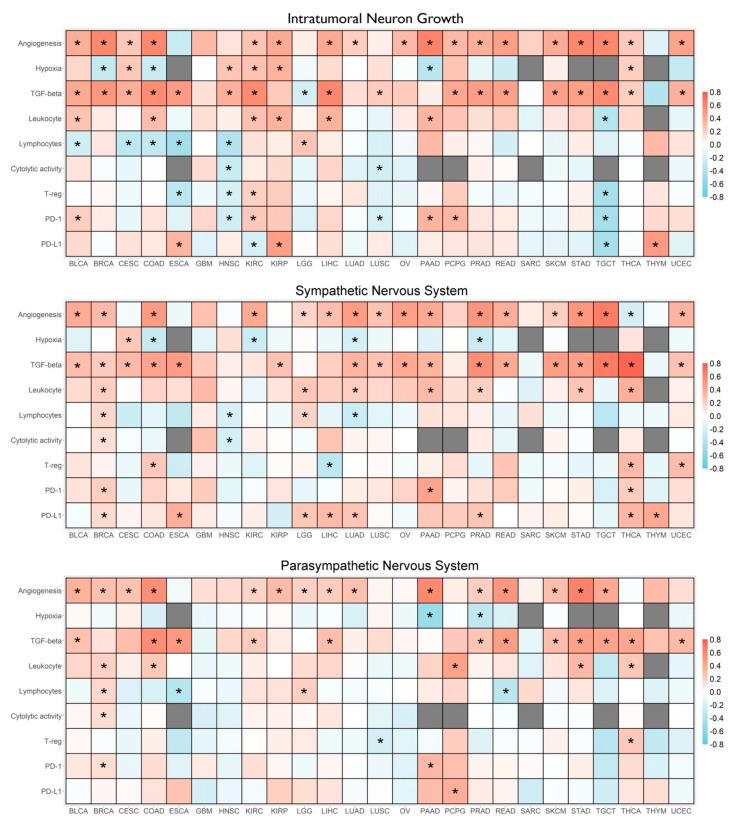
Correlations between autonomic nervous system gene expression signatures with tumor molecular and immunological characteristics. Asterisks indicate significant correlations after correcting for multiple comparison.

Correlations between ANS signatures and levels of selected molecular markers of angiogenesis, hypoxia, and TGF-β signaling, as well as immunological features in the TME, including the estimated proportion of leukocytes, lymphocytes, regulatory T-cells, cytolytic activity, and PD-1 and PD-L1 expression were calculated for each cancer type, and the results are summarized in Figure 2. All correlations were derived from the 25 cancer types, for which data were available from at least 100 cases. The TNG signatures were significantly and positively correlated with angiogenesis in 18 (72%) of the tested cancer types, with the strongest correlation found in pancreatic adenocarcinoma (PAAD) (r = 0.59, *p* < 1.0 × 10^−15^), and also associated with TGF-β pathway activation in 17 (68%) tested cancer types, with the strongest correlation found in liver hepatocellular carcinoma (LIHC) (r = 0.57, *p* < 1.0 × 10^−15^). Similar results were found for both SNS and PNS, with the strongest correlation between SNS and TGF-β in thyroid carcinoma (THCA) (r = 0.73, *p* < 1.0 × 10^−15^).

For cancer types with data available from at least 100 cases in TCGA, Pearson correlation coefficients were determined between each of our three autonomic nervous system (ANS) signatures and selected molecular and immunological characteristics. Correlation coefficients are color-coded according to direction and strength as indicated by the legend included on the right margin. Statistically significant correlations after correcting for multiple comparisons are indicated by an asterisk. TCGA cancer symbols are as follows: BLCA, bladder urothelial carcinoma; BRCA, breast invasive carcinoma; CESC, cervical squamous cell carcinoma and endocervical adenocarcinoma; COAD, colon adenocarcinoma; ESCA, esophageal carcinoma; GBM, glioblastoma multiforme; HNSC, head and neck squamous cell carcinoma; KIRC, kidney renal clear cell carcinoma; KIRP, kidney renal papillary cell carcinoma; LGG, brain low grade glioma; LIHC, liver hepatocellular carcinoma; LUAD, lung adenocarcinoma; LUSC, lung squamous cell carcinoma; OV, ovarian serous cystadenocarcinoma; PAAD, pancreatic adenocarcinoma; PCPG, pheochromocytoma and paraganglioma; PRAD, prostate adenocarcinoma; READ, rectum adenocarcinoma; SARC, sarcoma; SKCM, skin cutaneous melanoma; STAD, stomach adenocarcinoma; TGCT, testicular germ cell tumors; THCA, thyroid carcinoma; THYM, thymoma; UCEC, uterine corpus endometrial carcinoma.

Levels of ANS signatures and immunosuppression in the TME were found to be significantly correlated in six cancer types. The presence of lymphocytes was significantly lower in several tumors with higher levels of TNG signature, including bladder urothelial carcinoma (BLCA), cervical cancer (CESC), colon adenocarcinoma (COAD), esophageal carcinoma (ESCA), and HNSC. Moreover, decreased cytolytic activity was found in lung squamous cell carcinoma (LUSC), and HNSC and increased levels of T-regs were found in renal clear cell carcinoma (KIRC). The most common findings of immunosuppression may be due to the positive correlation of SNS signatures with PD-1 or PD-L1 expression, with significant associations in nine cancer types including BRCA, ESCA, LGG, LIHC, lung adenocarcinoma (LUAD), PAAD, PRAD, THCA, and thymoma (THYM).

### 3.3. Associations of ANS Signatures with Tumor Histopathological Features

**Figure 3:**
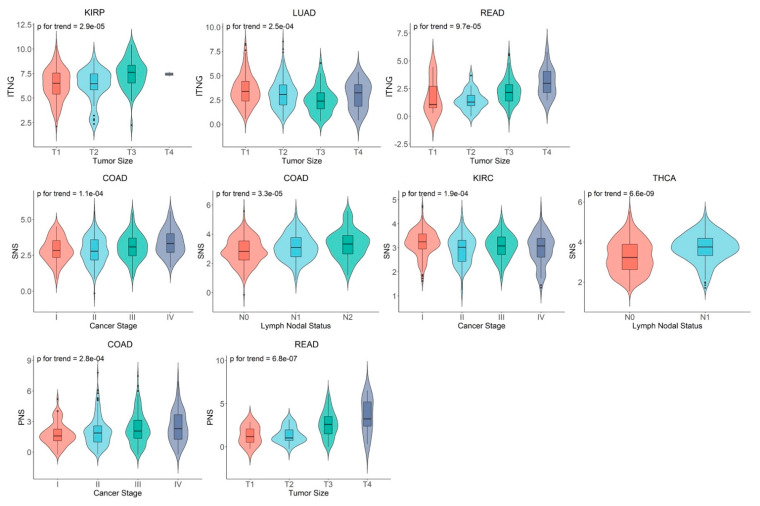
Autonomic nervous system gene expression signatures by tumor histopathological features.

Associations of selected histopathological variables, including cancer stage, tumor size, and lymph node status, were tested with each of the expression levels of the three ANS signatures. Stronger ANS signatures were associated with more advanced presentation in several cancer types. TNG signature levels were higher in larger tumors of renal papillary cell carcinoma (KIRP) (*p* = 2.9 × 10^−5^) and rectal adenocarcinoma (READ) (*p* = 9.7 × 10^−5^) but were lower in larger tumors of LUAD (*p* = 2.5 × 10^−4^). SNS signature levels were increased in tumors of higher stage (*p* = 1.1 × 10^−4^) and lymph node involvement (*p* = 3.3 × 10^−5^) in COAD, as well as with lymph node involvement in THCA (*p* = 6.6 × 10^−9^); however, SNS signature levels were lower in more advanced stages of KIRC (*p* = 1.9 × 10^−4^). Lastly, increased PNS levels were found in more advanced stages of COAD (*p* = 2.8 × 10^−4^) and larger tumors in READ (*p* = 6.8 × 10^−7^). These results are depicted in Figure 3. 

Violin plots are constructed for autonomic nervous system (ANS) signatures by selected tumor histopathological features for cancer types with data available from at least 100 cases in TCGA. Only cancer types for which *p*-values remained significant after correcting for multiple comparison are shown. TCGA cancer symbols are as follows: COAD, colon adenocarcinoma; KIRC, kidney renal clear cell carcinoma; KIRP, kidney renal papillary cell carcinoma; LUAD, lung adenocarcinoma; READ, rectum adenocarcinoma; THCA, thyroid carcinoma.

### 3.4. Associations of ANS Signatures with Patient Survival Outcomes

**Figure 4:**
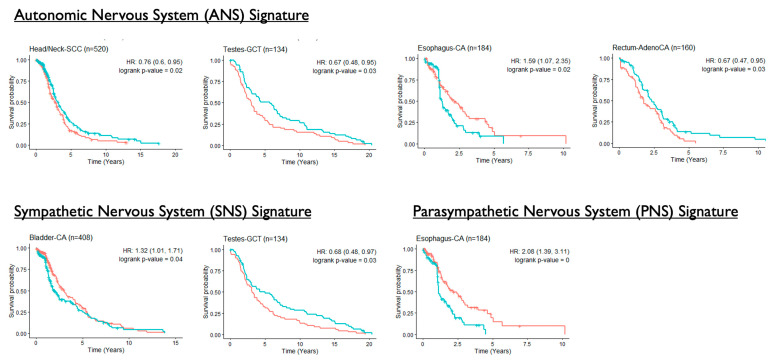
Kaplan–Meier curves of all-cause mortality by the levels of autonomic nervous system gene expression signatures. Red and blue lines represent low and high levels of signature levels, respectively, based on the median.

Kaplan–Meier curves were plotted for all-cause mortality where significant differences were found in several cancer types between the higher vs. lower levels of ANS signatures (Figure 4). In ESCA, higher levels of TNG and PNS were associated with higher mortality (TNG: HR = 1.59, 95% CI: 1.07–2.35; PNS: HR = 2.08, 95% CI: 1.39–3.11). A similar finding was noted for SNS signatures in BLCA (HR = 1.32, 95% CI: 1.01–1.71). On the contrary, higher levels of TNG were associated with lower mortality in HNSC (HR = 0.76, 95% CI: 0.60–0.95) and READ (HR = 0.67, 95% CI: 0.47–0.95). In TGCT, patients with higher levels of TNG (HR = 0.67, 95% CI: 0.48–0.95) and SNS (HR = 0.68, 95% CI: 0.48–0.97) had lower all-cause mortality.

Kaplan–Meier survival curves for all-cause mortality are plotted by the levels of the three autonomic nervous system signatures (high (red lines) vs. low (blue lines) level relative to median) for cancer types where data were available from at least 100 cases in TCGA. Log-rank *p*-values, hazard ratios (HRs), and 95% confidence intervals (CIs) are displayed. Only results from cancer types with a significant *p*-value ≤ 0.05 are shown.

## 4. Discussion

Our study surveyed the expression levels of three ANS gene signatures to determine relative levels of intratumoral autonomic nerve infiltration in 32 cancer types. We found wide variability both within and across cancer types in the associations of ANS signatures with tumor histopathological characteristics and survival outcomes. Further, our pan-cancer analyses revealed three molecular and immunological correlates with ANS expression that were common across cancer types, including angiogenesis, TGF-β signaling, and immunosuppression (measured as TME levels of PD-1, PD-L1, and T-regs). While previous studies over the past two decades have increasingly demonstrated the role that intratumoral autonomic innervation plays in cancer progression, it has only recently been shown that β-adrenergic signaling might be the link between chronic psychosocial stress and increased cancer aggressiveness and patient mortality [3]. Such findings suggest that commonly prescribed pharmaceutical therapies such as beta-blockers could be used to improve cancer patient outcomes, though the extent of autonomic innervation in specific cancer types for which such therapy may be of benefit has previously been poorly understood.

Various studies have shown an association between autonomic innervation and increased tumor angiogenesis, but the underlying pathophysiology had for a long time remained uncertain. Zahalka et al. in 2017 demonstrated that sympathetic inputs into the TME increase local levels of norepinephrine and activate ADRβ2 receptors on the surface of vascular endothelial cells, resulting in a downstream suppression of oxidative phosphorylation [2]. The overall effect of this “metabolic switch” is an increase in anaerobic metabolism, which promotes the production of hypoxic and vasogenic factors that facilitate intratumoral angiogenesis [2]. The results of our study corroborate Zahalka et al.’s immunohistological findings, as we observed that increased ANS innervation was significantly correlated with higher levels of intratumoral angiogenesis in 18 of our 25 (72%) tested cancer types. This mechanism by which autonomic innervation mediates vascular endothelial growth factor (VEGF)-driven angiogenesis may also explain why pharmacological inhibition of the VEGF pathway with targeted agents, such as Bevacizumab and Sorafenib, have shown variable utility in the prevention of cancer progression. While anti-VEGF agents have demonstrated efficacy in the treatment of renal cell, hepatocellular, colorectal, non-small cell lung, and ovarian cancers, such agents have shown only modest effects on long-term prognosis in breast, pancreatic, and prostate cancers [14]. In breast cancer specifically, ADRβ2 signaling increases the expression of VEGF by breast cancer cells via upregulation of Notch and Jagged 1 mRNA translation in response to increased levels of norepinephrine in the local environment [15]. These angiogenic effects were observed to be inducible with use of salmeterol (an ADRβ2 agonist) and reversible with the use of propranolol (a nonselective β-adrenergic antagonist) [15], suggesting that in certain cancer types, antiadrenergic treatment may show promise in slowing cancer progression either as monotherapy or in combination with anti-VEGF agents.

TGF-β signaling has been found to have both antitumoral and tumorigenic properties. While TGF-β suppresses cell proliferation and induces apoptosis in healthy and premalignant cells [16], Korkut et al. demonstrated that dysregulation of genes involved in the TGF-β signaling pathway has been implicated in over 50% of tissue samples from 12 different cancer types [17]. Elevated levels of TGF-β have been found in advanced stages of breast, lung, hepatocellular, and prostate cancers [18], and correlated with increased tumor aggressiveness and poorer clinical outcomes [19]. TGF-β signaling promotes cancer metastasis through its induction of epithelial–mesenchymal transition, facilitated by increased expression of various matrix metalloproteases [20] and disruption of epithelial cell adhesion via downregulation of E-cadherin expression [21]. In addition, angiogenic mediators such as VEGF [22] and connective tissue growth factor (CTGF) [23] have been shown to be downstream targets of TGF-β-mediated SMAD activation, suggesting TGF-β also has a role in promoting intratumoral angiogenesis. TGF-β released by tumor cells into the TME also decreases the effectiveness of natural killer (NK) and CD8+ T-cells to eliminate cancer cells through the downregulation of interleukin-2 production [24,25]. Our study found increased TGF-β pathway levels correlated with increased ANS expression in 17 of 25 (68%) cancer types, suggesting a potentially novel role for this signaling pathway in promoting tumor progression through intratumoral innervation, and warranting further mechanistic investigation in the future.

Increased autonomic signaling has been shown to produce a local immunosuppressive TME by recruiting and increasing the immunosuppressive function of myeloid-derived suppressor cells (MDSCs) [26,27], inhibiting T-cell metabolic fitness [28], T-cell proliferation, cytokine production, and cytotoxicity [29,30]. This process is thought to be mediated by increased expression of PD-1 and PD-L1, along with direct activation of β-adrenergic receptors on the surface of infiltrating CD8+ T-cells and resultant disruption of CD28 co-stimulation with antigen-presenting cells [29]. PD-L1 signaling promotes immunosuppression by binding to PD-1 receptors on CD8+ T-cells and inducing immune tolerance and decreased lymphocyte migration to the tumor site [31]. Mo et al. found that PD-L1 was highly expressed on infiltrating prostate TME-associated autonomic fibers, but rarely found on prostate cancer cells themselves [32], suggesting that autonomic nerves are directly responsible for the production of an immunosuppressive TME. The increased density of autonomic innervation has also been significantly associated with cancer recurrence and poorer long-term outcome, attributed to a weakened antitumor immunity caused by decreased CD8 T-lymphocyte levels [32]. PD-L1 has also been shown to have an important role in the activation and maintenance of T-regs [33], and increased expression of PD-L1 in the TME has been correlated with increased T-reg levels and decreased immune surveillance. T-regs normally are involved in immunosuppressive responses in healthy individuals where they play a role in preventing the development of autoimmune disorders, but T-regs have also been shown to be recruited by cancer cells to create localized immunosuppressive environments [34]. It has been suggested that activation of β-adrenergic receptors on T-regs via sympathetic innervation of the TME is able to increase the immunosuppressive functions of T-regs [35]. Our study found levels of T-regs were significantly elevated in colon, thyroid, and uterine cancers with increased ANS innervation. Conversely, T-regs were also found to be significantly decreased in esophageal cancer, head and neck squamous cell carcinoma, and testicular germ cell tumors with increased ANS innervation.

Although our study found that increased ANS innervation was correlated with significant reductions in intratumoral lymphocyte levels in bladder, cervical, colon, esophageal, and head and neck cancers, ANS innervation was also correlated with significantly elevated leukocyte levels in 13 other cancer types. Testicular germ cell tumor was the only cancer type in our study which demonstrated significant reductions in leukocyte proportions with increasing ANS expression. This finding is likely explained by previous studies that have shown that, while β-adrenergic signaling disrupts T-cell proliferation and migration, the ongoing anti-tumor inflammatory response produces large amounts of cytokines and chemotactic factors that increase capillary permeability and intratumoral migration of other leukocyte types [36]. β-adrenergic signaling has specifically been shown to increase macrophage recruitment to the TME, which subsequently stimulates the release of various factors that promote cancer progression, including TGF-β, VEGF, interleukin-6, matrix metalloproteinase-9 (MMP-9), and prostaglandin-endoperoxide synthase 2 (PTGS2) [37]. Tumor cell production of monocyte chemotactic protein-1 (MCP-1) [38] and VEGF-A [39] results in the recruitment of monocytes and macrophages into the TME, which has been associated with increased tumor invasion and poorer cancer prognosis [40].

While our study demonstrated significant correlations between ANS innervation and increased angiogenesis, TGF-β levels, and immunosuppression in many cancer types, we did not observe a strong association with survival outcome in most of our tested cancer types. Esophageal and bladder carcinoma demonstrated higher mortality in patients with increased intratumoral ANS innervation, while, paradoxically, squamous cell carcinoma of the head and neck, testicular germ cell tumor, and rectal adenocarcinoma showed improved survival with higher levels of ANS innervation. These results support Kim et al.’s previous finding that beta-blocker use was associated with decreased survival in patients with squamous cell carcinoma of the head and neck [41]; however, we also showed a positive survival association between ANS innervation and rectal adenocarcinoma, unlike Hicks et al.’s study suggesting no association [42]. In many of the rarer cancer types we studied, we hypothesize the lack of associations between ANS innervation and cancer mortality may be related to small sample sizes and short follow-up times. However, our data demonstrate that the impact of intratumoral ANS innervation on patient survival outcomes is dependent on cancer type.

The involvement of β-adrenergic signaling in cancer progression suggests that suppression of this pathway with the use of beta-blockers may improve cancer outcomes. Epidemiological studies have shown that incidental treatment of cancer patients with beta-blockers improves patient outcomes in cases of esophageal [43], breast [44], prostate [45,46], ovarian [47], non-small cell lung [48], and stage IV colorectal [49] cancers, though the evidence for overall treatment efficacy has been conflicting due to null findings in other studies [50]. A meta-analysis by Yap et al. of cancer recurrence and mortality rates in patients who took beta-blockers demonstrated improvements in patient survival rates in cases of melanoma and ovarian cancer, but worsened outcomes in endometrial, prostate, head and neck, and lung cancers [51]. The heterogeneous responses of cancer patients to beta-blockers may be attributed to variable β-adrenergic receptor expression levels across different cancer types. In an immunohistochemical study conducted by Rains et al. which examined the expression of β-adrenergic receptors in 389 tissues from 29 cancer types, β-adrenergic receptor staining exhibited the highest expression levels in esophageal, pancreatic, kidney, and lung cancers and melanoma compared to other cancer types [52]. However, the sample sizes for each cancer type in their study were limited, and no clinical correlates or patient outcomes were analyzed. The findings of our study support these conclusions and provide more evidence for the underlying physiological mechanisms responsible for the heterogeneity of patient outcomes and responses to beta-blocker therapy dependent on cancer type.

Because this was an in silico analysis based on TCGA data, and the marker genes for ANS signatures were selected from the current literature, future studies are warranted when newer gene-expression-based ANS signatures are developed and data are available from the cancer types with limited sample sizes in TCGA. Our study represents an early-phase investigation of intratumoral ANS as a potential therapeutic target for cancer treatment, and further preclinical and clinical studies will be needed before the potential can be translated for clinical utility.

In conclusion, our pan-cancer analysis based on TCGA data found cancer type-specific patterns of intratumoral ANS expression, with variable effects on tumor molecular, immunological, and histopathological characteristics and outcomes. We showed that intratumoral ANS expression was correlated with angiogenesis, TGF-β activation, and immunosuppression in the TME, providing mechanistic insights into the involvement of intratumoral innervation in cancer development and progression. Our findings suggest that the potential therapeutic benefits of targeting β-adrenergic receptor-mediated stress signaling on patient outcomes are likely cancer-type dependent.

## Data Availability

The data presented in this study are available on request from the corresponding author.

## Abbreviations

Manuscript Terms	
ADRβ2	β2-Adrenergic Receptor
ANS	Autonomic Nervous System
bFGF	Basic Fibroblast Growth Factor
cAMP	Cyclic Adenosine Monophosphate
CNS	Central Nervous System
CTGF	Connective Tissue Growth Factor
MMP	Matrix Metalloproteinase
NGF	Nerve Growth Factor
NK	Natural Killer
NT3	Neurotrophin-3
NT4/5	Neurotrophin-4/5
PNS	Parasympathetic Nervous System
SNS	Sympathetic Nervous System
TCGA	The Cancer Genome Atlas
TME	Tumor Microenvironment
VEGF	Vascular Endothelial Growth Factor
Cancer Types	
ACC	Adrenocortical Carcinoma
BLCA	Bladder Urothelial Carcinoma
BRCA	Breast Invasive Carcinoma
CESC	Cervical Squamous Cell carcinoma and Endocervical Adenocarcinoma
CHOL	Cholangiocarcinoma
COAD	Colon Adenocarcinoma
DLBC	Diffuse Large B-cell Lymphoma
ESCA	Esophageal Carcinoma
GBM	Glioblastoma Multiforme
HNSC	Head and Neck Squamous Cell Carcinoma
KICH	Kidney Chromophobe
KIRC	Kidney Renal Clear Cell Carcinoma
KIRP	Kidney Renal Papillary Cell Carcinoma
LGG	Brain Lower Grade Glioma
LIHC	Liver Hepatocellular Carcinoma
LUAD	Lung Adenocarcinoma
LUSC	Lung Squamous Cell Carcinoma
MESO	Mesothelioma
OV	Ovarian Serous Cystadenocarcinoma
PAAD	Pancreatic Adenocarcinoma
PCPG	Pheochromocytoma and Paraganglioma
PRAD	Prostate Adenocarcinoma
READ	Rectum Adenocarcinoma
SARC	Sarcoma
SKCM	Skin Cutaneous Melanoma
STAD	Stomach Adenocarcinoma
TGCT	Testicular Germ Cell Tumors
THCA	Thyroid Carcinoma
THYM	Thymoma
UCEC	Uterine Corpus Endometrial Carcinoma
UCS	Uterine Carcinosarcoma
UVM	Uveal Melanoma
